# FOLFOX regimen after failure of fluorouracil and leucovorin plus nanoliposomal-irinotecan therapy for advanced pancreatic cancer: a retrospective observational study

**DOI:** 10.1186/s12885-023-10654-3

**Published:** 2023-02-21

**Authors:** Satoshi Kobayashi, Shun Tezuka, Yui Yamachika, Shotaro Tsunoda, Shuhei Nagashima, Yuichiro Tozuka, Taito Fukushima, Manabu Morimoto, Makoto Ueno, Junji Furuse, Shin Maeda

**Affiliations:** 1 Department of Gastroenterology, Kanagawa Cancer Center, 2-3-2, Nakao, Asahi-ku, Yokohama City, Kanagawa 241-0815 Japan; 2 Department of Gastroenterology, Yokohama City University Graduate School of Medicine, 3-9, Fukuura, Kanazaw-ku, Yokohama City, Kanagawa 236-0004 Japan

**Keywords:** Oxaliplatin, FOLFOX, Third-line treatment, Salvage, CRP, Homologous recombination deficiency

## Abstract

**Background:**

Fluorouracil, leucovorin (5FU/LV), and nanoliposomal-irinotecan (nal-IRI) combination therapy has been established as the second-line treatment for advanced pancreatic ductal adenocarcinoma. Oxaliplatin with 5FU/LV (FOLFOX) is often used as a subsequent treatment, although its efficacy and safety are yet to be fully elucidated. We aimed to evaluate the efficacy and safety of FOLFOX as a third- or later-line treatment for patients with advanced pancreatic ductal adenocarcinoma.

**Methods:**

We conducted a single-centre, retrospective study that enrolled 43 patients who received FOLFOX after failure of gemcitabine-based regimen followed by 5FU/LV + nal-IRI therapy between October 2020 and January 2022. FOLFOX therapy consisted of oxaliplatin (85 mg/m^2^), levo-leucovorin calcium (200 mg/m^2^) and 5-FU (2400 mg/m^2^) every 2 weeks per cycle. Overall survival, progression-free survival, objective response, and adverse events were evaluated.

**Results:**

At the median follow-up time of 3.9 months in all patients, the median overall survival and progression-free survival were 3.9 months (95% confidence interval [CI], 3.1–4.8) and 1.3 months (95% CI, 1.0–1.5), respectively. Response and disease control rates were 0 and 25.6%, respectively. The most common adverse event was anaemia in all grades followed by anorexia; the incidence of anorexia and grades 3 and 4 was 21 and 4.7%, respectively. Notably, grades 3–4 peripheral sensory neuropathy was not observed. Multivariable analysis revealed that a C-reactive protein (CRP) level of > 1.0 mg/dL was a poor prognostic factor for both progression-free survival and overall survival: hazard ratios were 2.037 (95% CI, 1.010–4.107; *p* = 0.047) and 2.471 (95% CI, 1.063–5.745; *p* = 0.036), respectively.

**Conclusion:**

FOLFOX as a subsequent treatment after failure of second-line treatment with 5FU/LV + nal-IRI is tolerable, although its efficacy is limited, particularly in patients with high CRP levels.

**Supplementary Information:**

The online version contains supplementary material available at 10.1186/s12885-023-10654-3.

## Background

Pancreatic ductal adenocarcinoma (PDAC) is the seventh leading cause of cancer-related death worldwide, accounting for approximately 466,000 cases in 2020 [1]. Surgical resection is the only curative treatment; however, PDACs are generally detected at an unresectable stage. Therefore, the efficacy of various systemic chemotherapies has been evaluated. Recently, several combination regimens have been developed for advanced PDAC, including the FOLFIRINOX regimen which is composed of fluorouracil (5-FU), leucovorin (LV), irinotecan, and oxaliplatin (L-OHP) [2], as well as gemcitabine (GEM) plus nab-paclitaxel (nab-PTX) [3]. Hence, the treatment guidelines of the National Comprehensive Cancer Network and the Japanese Society of Pancreas recommend the FOLFIRINOX regimen and GEM + nab-PTX as first-line treatment for patients with unresectable PDAC [4, 5].

Regarding second-line treatment after a GEM-based regimen, 5-FU/LV plus nanoliposomal-irinotecan (nal-IRI) showed superior overall survival (OS) to 5-FU/LV in a phase III NAPOLI-1 trial [6]. In addition, a phase II study in Japan comparing the efficacies of 5-FU/LV plus nal-IRI and 5-FU/LV therapies showed results consistent with those of the NAPOLI-1 trial [7]; treatment with 5-FU/LV plus nal-IRI was approved in Japan in 2020. Although a second-line treatment was established, the median progression-free survival (PFS) and time-to-treatment failure were 3.1 months and 2.3 months, respectively, in the NAPOLI-1 trial; results in clinical settings were similar with a median of PFS of 4.2 months [8]. Therefore, most patients require subsequent treatment after 5-FU/LV plus nal-IRI.

Based on the results of the CONKO-003 trial, L-OHP may have anticancer activity in PDAC refractory to gemcitabine-based regimens [9], and patients with a prior history of GEM+nab-PTX and 5-FU/LV + nal-IRI are candidates for 5-FU/LV plus L-OHP (FOLFOX) treatment. In contrast, neither 5-FU/LV plus L-OHP nor S-1 (oral fluoropyrimidine drug) plus L-OHP showed efficacy as second-line treatments for advanced PDAC in each clinical trial [10, 11]. There remains a controversy regarding the efficacy of FOLFOX as a second-line treatment, while data regarding FOLFOX as a third- or later-line treatment are limited. Therefore, we aimed to evaluate the efficacy and safety of FOLFOX as a third- or later-line treatment for patients with advanced PDAC and explore the prognostic factors for PFS along with OS.

## Methods

### Patients

This single-centre retrospective study enrolled 43 consecutive patients who were started on the FOLFOX regimen after failure of the GEM-based regimen and 5-FU/LV plus nal-IRI at the Kanagawa Cancer Centre between October 2020 and January 2022. We introduced FOLFOX if patients had an Eastern Cooperative Oncology Group performance status (ECOG PS) of 0–2; were 80 years or younger; were capable of consuming an adequate amount of food; had a peripheral sensory neuropathy grade of 0–2; had normal liver, renal, and bone marrow functions. Adjuvant chemotherapy was excluded from the number of prior chemotherapy histories, even if the disease recurred during or after completion of adjuvant chemotherapy. Cancer genome information, including *BRCA* mutations, was evaluated, if applicable.

### Treatment

The FOLFOX regimen consisted of L-OHP at a dose of 85 mg/m^2^ delivered via a 90-min intravenous infusion, immediately followed by levo-leucovorin calcium at a dose of 200 mg/m^2^ delivered via a 60-min intravenous infusion, followed by 5-FU at a dose of 2400 mg/m^2^ delivered via a 46-h continuous intravenous infusion. The initial dose was reduced in some patients at the physician’s discretion. Treatment was repeated once biweekly and continued until disease progression, unacceptable adverse events, or patient refusal.

### Treatment outcomes

We evaluated OS, PFS, and objective radiological response as efficacy endpoints and adverse events during FOLFOX treatment as a safety endpoint. OS was defined as the time from the date of FOLFOX initiation to that of death from any cause, while PFS was defined as the time from the date of FOLFOX initiation to that of documented disease progression or death from any cause. We treated patients as censored cases that did not show any events related to OS or PFS. Objective response was classified according to the Response Evaluation Criteria in Solid Tumour version 1.1, in which the response was classified into complete response (CR), partial response (PR), stable disease (SD), and progressive disease (PD) [12]. Objective response rate was defined as the number of patients who showed CR and PR divided by the total number of patients. The disease control rate was defined as the number of patients who showed CR, PR, and SD divided by the total number of patients. Adverse events were evaluated according to the Common Terminology Criteria for Adverse Events, version 5 [13].

### Statistical analysis

Categorical values are expressed as the number of patients and their percentages. Continuous values are expressed as medians with ranges. Comparisons of categorical and continuous values between some subgroups were conducted using Fisher’s exact test and the Mann-Whitney *U* test, respectively. The median OS and PFS were estimated using the Kaplan-Meier method. OS and PFS were compared between subgroups using the log-rank test.

To explore factors contributing to poorer PFS and OS, we conducted multivariable analysis using Cox regression analysis with covariates with a *p*-value of < 0.2 in univariate analysis. All statistical analyses were conducted using SPSS Statistics version 23 (IBM SPSS, Inc., Chicago, IL, USA). Statistical significance was considered at *p*-value of < 0.05.

## Results

### Patients

Patient background data are listed in Table 1. The median age was 67 years, and 30 patients were male. Eleven patients (25.6%) had an ECOG PS of 0, whereas the rest had an ECOG PS of 1. FOLFOX was administered as the third-, fourth-, and fifth- lines of treatment in 34, 5, and 4 patients, respectively. Baseline haemoglobin level was 11.4 g/dL in median, while grades 1 and 2 anaemia were observed in 12 patients (27.9%) each. Among the 34 patients whose germline *BRCA* status was tested, only 1 had a mutation (2.9%). The remaining nine patients were not tested for *BRCA* mutations due to their general condition or older age.

**Table 1:** Patient characteristics

Factor	N (%)
Age (years)	
median (range)	67 (48–84)
Sex	
Male	30 (69.8)
Female	13 (30.2)
ECOG PS^a^	
0	11 (25.6)
1–2	32 (74.4)
Disease status	
Metastatic	34 (79.1)
Recurrence	9 (20.9)
Treatment line	
Third line	34 (79.1)
Fourth line	5 (11.6)
Fifth line	4 (9.3)
CEA^b^ (ng/mL)	
median (range)	11.6 (2.5–669.7)
CA 19–9^c^ (U/mL)	
median (range)	2762.7 (0–115,360.2)
CRP^d^ (mg/dL)	
median (range)	1.46 (0.03–10.0)
Haemoglobin (g/dL)	
median (range)	11.4 (13.9–8.1)
Albumin (g/dL)	
median (range)	3.5 (2.2–4.2)
Germline BRCA mutation	
Positive	1 (2.3)
Negative	30 (76.7)
Not assessed	9 (20.9)
Reason for discontinuation of 5-FU/LV + nal-IRI^e^	
Progression	42 (97.7)
Intolerable toxicity	1 (2.3)

^a^ *ECOG PS* Eastern Cooperative Oncology Group performance status

^b^ *CEA* Carcinoembryonic antigen

^c^ *CA 19–9* Carbohydrate antigen 19–9

^d^ *CRP* C-reactive protein

^e^ *5-FU/LV + nal-IRI* Fluorouracil, leucovorin, and nanoliposomal-irinotecan combination therapy

#### Treatment

The initial dose reduction of L-OHP, 5-FU, or both L-OHP and 5-FU was employed in four (9.3%), eight (18.6%), and seven (16.3%) patients, respectively. Additionally, 7 (16.3%) and 10 (23.3%) patients required dose reduction of each agent during treatment, according to adverse events. All patients discontinued the FOLFOX regimen at the time of data cut-off. The reason for discontinuation was disease progression in 41 patients (95.3%) and adverse events (grade 3 anaphylactic reaction and prolonged platelet count decrease of grade 3) in 1 patient for each (4.7%).

#### Efficacy

**Fig. 1:**
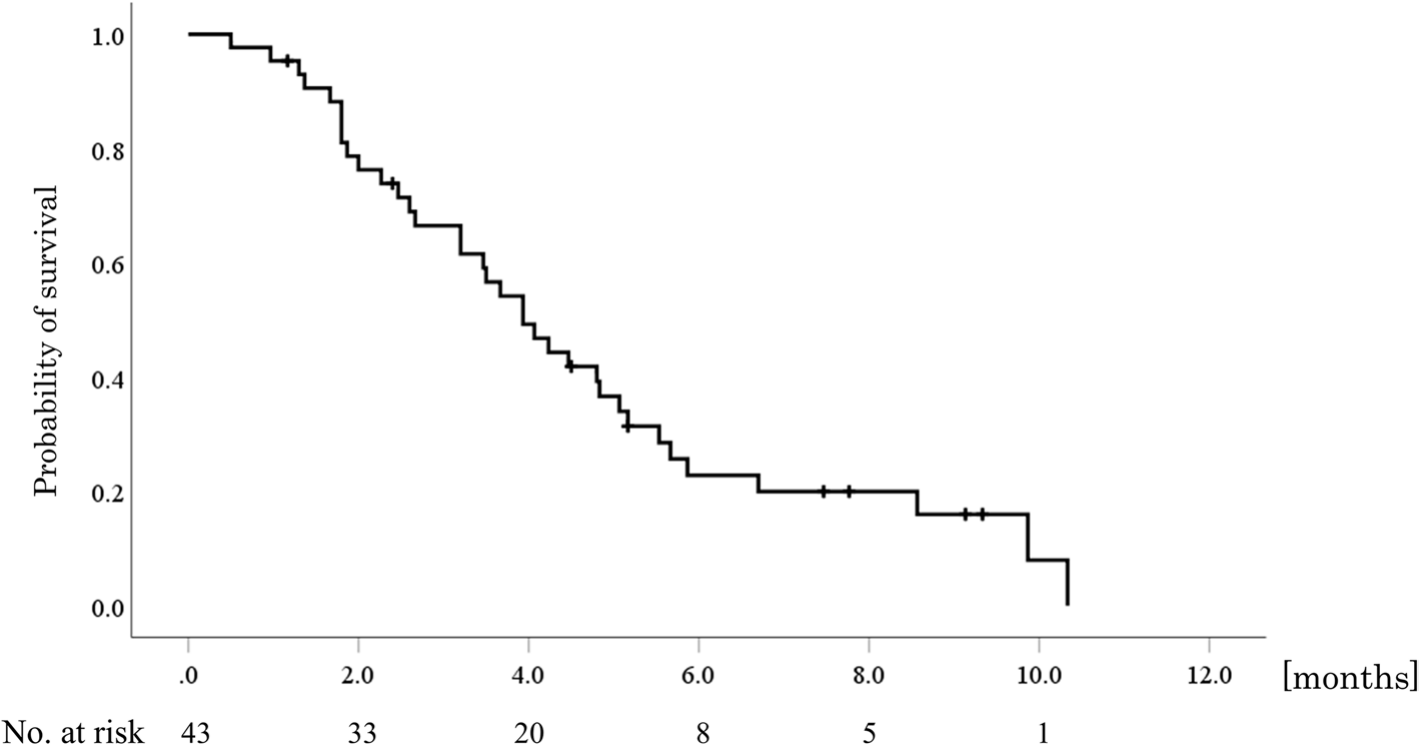
Kaplan-Meier curve of overall survival. The median overall survival was 3.9 months (95% confidence interval, 3.1–4.8)

**Fig. 2:**
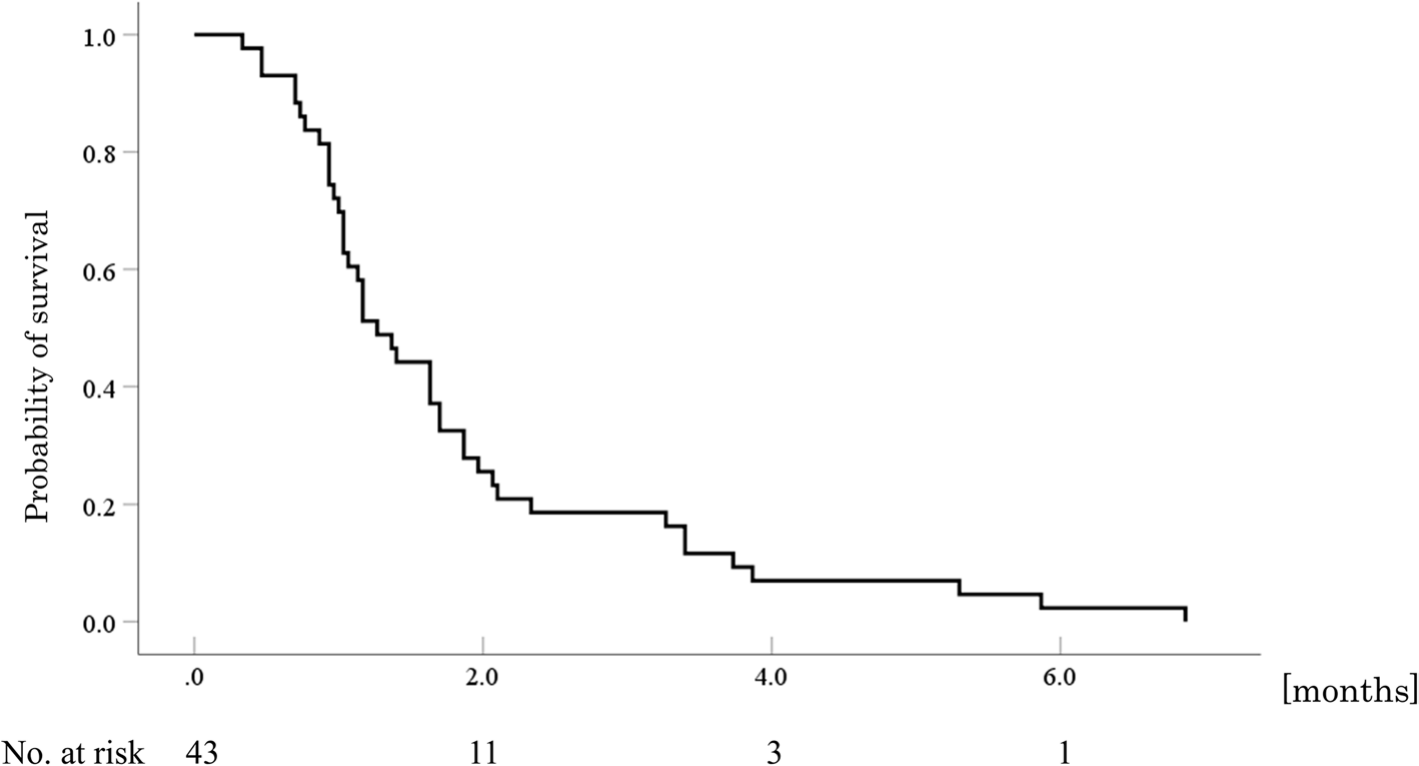
Kaplan-Meier curve of progression-free survival. The median progression-free survival was 1.3 months (95% confidence interval, 1.0–1.5)

At a median follow-up time of 3.9 months in all patients and 6.3 months in the censored patients, the median OS was 3.9 months (95% confidence interval [CI], 3.1–4.8) (Fig. 1) and median PFS was 1.3 months (95% CI, 1.0–1.5) (Fig. 2). No patient showed PR, and SD was observed in 11 patients, corresponding to an objective response rate of 0% and disease control rate of 25.6%.

#### Safety

The overall toxicity of the regimen was tolerable (Table 2). The most common adverse event was anaemia, which accounted for 74% of all grades; however, most were observed at baseline and no case of grade ≥ 3 anaemia was observed (see additional fig. 1). Among non-haematological adverse events, anorexia was the most common, accounting for 21% in all grades and 4.7% in grade 3 or higher. Notably, peripheral sensory neuropathy of grade ≥ 3 was not observed and considered mild.

**Table 2:** Adverse events

Terms	N (%)			
	Grade 1	Grade 2	Grade 3	Grade 4
Anaemia	19 (44.2)	13 (30.2)	0	0
Neutropenia	4 (9.3)	8 (18.6)	3 (7.0)	0
Thrombocytopenia	9 (21.0)	3 (7.0)	0	0
Febrile neutropenia	–	–	0	0
Anorexia	4 (9.3)	3 (7.0)	2 (4.7)	0
Nausea	0	4 (9.3)	0	0
Vomiting	0	2 (4.7)	2 (4.7)	0
Peripheral sensory neuropathy	0	4 (9.3)	0	0
Diarrhoea	0	3 (7.0)	0	0
Malaise	1 (2.3)	0	1 (2.3)	0

#### Exploratory analysis of prognostic factor

Univariate analysis of factors contributing to PFS showed that ECOG PS, treatment line, and serum levels of CEA, CA 19–9, and CRP had *p*-values of < 0.2. Multivariable analysis revealed that only a serum CRP level >  1.0 mg/dL was a significant poor prognostic factor for PFS (hazard ratio, 2.037; 95% CI, 1.010–4.107; *p* = 0.047) (Table 3).

**Table 3:** Prognostic factors for progression-free survival

	N (%)	Univariate analysis	Multivariable analysis		
		*P*-value	hazard ratio	95% CI^e^	*P*-value
Age: > 70 (vs. ≤ 70)	19 (44)	0.454			
Sex: female (vs. male)	13 (30)	0.447			
ECOG PS^a^: 1–2 (vs. 0)	32 (74)	0.189	1.450	0.523–4.026	0.475
Disease status: recurrence (vs. metastatic)	9 (21)	0.222			
Treatment line: 4th or later (vs. 3rd)	9 (21)	0.112	0.503	0.205–1.239	0.135
CEA^b^(ng/mL): > 10.0 (vs. ≤ 10.0)	23 (53)	0.012	1.958	0.845–4.537	0.117
CA 19–9^c^ (U/mL): > 2000 (vs. ≤ 2000)	24 (56)	0.122	0.434	0.651–2.720	0.434
Albumin (g/dL): ≤ 3.5 (vs. > 3.5)	24 (56)	0.663			
CRP^d^ (mg/dL): > 1.0 (vs. ≤ 1.0)	16 (37)	0.021	2.037	1.010–4.107	0.047

^a^ *ECOG PS* Eastern Cooperative Oncology Group performance status

^b^ *CEA* Carcinoembryonic antigen

^c^ *CA 19–9* Carbohydrate antigen 19–9

^d^ *CRP* C-reactive protein

^e^ *CI* Confidence interval

Univariate analysis of factors contributing to OS showed that sex and serum levels of albumin and CRP had *p*-values of < 0.2. Multivariable analysis revealed that female sex and serum CRP levels > 1.0 mg/dL were significant poor prognostic factors for OS, with hazard ratios of 2.325 (95% CI, 1.056–5.118; *p* = 0.036) and 2.471 (95% CI, 1.063–5.745; *p* = 0.036), respectively (Table 4).

**Table 4:** Prognostic factors for overall survival

Factor	N (%)	Univariate analysis	Multivariable analysis		
		*P*-value	hazard ratio	95% CI^e^	*P*-value
Age: > 70 (vs. ≤ 70)	19 (44)	0.535			
Sex: female (vs. male)	13 (30)	0.020	2.325	1.056–5.118	0.036
ECOG PS^a^: 1–2 (vs. 0)	32 (74)	0.961			
Disease status: recurrence (vs. metastatic)	9 (21)	0.446			
Treatment line: 4th or later (vs. 3rd)	9 (21)	0.319			
CEA^b^(ng/mL): > 10.0 (vs. ≤ 10.0)	23 (53)	0.344			
CA 19–9^c^ (U/mL): > 2000 (vs. ≤ 2000)	24 (56)	0.228			
Albumin (g/dL): ≤ 3.5 (vs. > 3.5)	24 (56)	0.082	1.158	0.452–2.964	0.760
CRP^d^ (mg/dL): > 1.0 (vs. ≤ 1.0)	16 (37)	0.009	2.471	1.063–5.745	0.036

^a^ *ECOG PS* Eastern Cooperative Oncology Group performance status

^b^ *CEA* Carcinoembryonic antigen

^c^ *CA 19–9* Carbohydrate antigen 19–9

^d^ *CRP* C-reactive protein

^e^CI, confidence interval

## Discussion

Second-line treatment following a GEM-based regimen has been established based on the results of the NAPOLI-1 phase III trial [6]. Despite recent advances in treatment for patients with unresectable PDAC, the prognosis of these patients remains poor. Therefore, the development of subsequent treatments is urgently required. Currently, in Japan, L-OHP-containing regimens are sometimes used as a subsequent treatment after failure of the GEM-based regimen and 5-FU/LV + nal-IRI; however, their efficacy and safety have not been elucidated. Therefore, in this study, we evaluated the efficacy and safety of the FOLFOX regimen as third- or later-line treatment after the 5-FU/LV + nal-IRI regimen in 43 patients. Although the toxicity was acceptable, the efficacy was limited: the median PFS was only 1.3 months, and the disease control rate was only 25.6% with no PR.

The combination treatment of 5-FU, LV, and L-OHP has been tested as a second-line treatment after a GEM-based regimen in two previous phase III trials. The first trial was the CONKO-003 trial, which demonstrated improvement in OS in combination treatment of the three drugs (OFF regimen) compared with in 5-FU plus LV (FF) [9]: the median of OS and time to progression was 5.9 months and 2.9 months in the OFF arm, and 3.3 months and 2.0 months in the FF arm, respectively. Despite the statistical significance, the OFF regimen was inconvenient because 5-FU and LV were administered once a week via 24-h intravenous infusion. Therefore, a second trial, called the PANCREOX trial, was conducted. This trial employed a modified FOLFOX6 regimen in which 5-FU, LV, and L-OHP were administered biweekly via 48-h intravenous infusion [10]. Although this trial did not meet the primary endpoint of PFS, it showed that the median PFS was 3.1 months in the modified FOLFOX6 arm compared with the 2.9 months in the 5-FU/LV arm. Based on these results, the median PFS may be approximately 3 months when the combination of 5-FU, LV and L-OHP is used as a second-line treatment. We used the FOLFOX regimen after failure of 5-FU/LV + nal-IRI, as all patients except one who quit prior treatment due to adverse events would be resistant to 5-FU/LV. Hence, it is reasonable that the median PFS in our study was worse than that in studies on second-line treatment. Yamai et al. also evaluated the efficacy of the FOLFOX regimen as salvage treatment after GEM plus nab-PTX and 5-FU/LV + nal-IRI [14]; although the sample size was 17, which was smaller than that in our study, the results are consistent with those of our study.

The continuous use of 5-FU/LV after failure is an issue that needs to be discussed. We considered 5-FU as a key drug for advanced pancreatic cancer because the standard treatment of PDAC in every disease stage includes 5-FU: GEM+S-1 as a neoadjuvant therapy for resectable disease [15], GEM+capecitabine [16], FOLFIRINOX [17] and S-1 [18] in adjuvant therapy after radical resection, and FOLFIRINOX in borderline and unresectable stages [2, 19, 20]. Therefore, we used 5-FU/LV with L-OHP even after failure of the 5-FU/LV containing regimen rather than L-OHP monotherapy. In contrast, L-OHP monotherapy was selected in most patients who received subsequent therapy in the FF arm of CONKO-003 [9]; this might be because they considered that continuous use of 5-FU/LV was not effective and that L-OHP monotherapy could maintain efficacy and improve safety. Indeed, we did not reduce the initial dose of L-OHP but reduced that of 5-FU in eight patients (18.6%). In addition, the efficacy results of a phase II study of L-OHP monotherapy as a second-line treatment for advanced PDAC were comparable with those of our study: SD for more than 2 months and the clinical benefit response was observed in 16.7 and 27.7% of patients, respectively [21]. Taking these results into consideration, continuous use of 5-FU after failure might be ineffective for advanced PDAC, although further investigation using a randomised controlled study is needed.

Although the overall efficacy of FOLFOX was insufficient to consider a standard treatment, multivariable analysis showed that patients with serum CRP < 1.0 mg/dL had better PFS and OS than those with ≥1.0 mg/dL. This indicates that patients with low CRP levels might be a good indication for FOLFOX, even in the third or later lines. Serum CRP level has often been reported as a prognostic factor for OS in advanced PDAC [22–24]. Haas et al. reported that serum CRP levels had the highest hazard ratio for OS among CEA, CA 19–9, and LDH in patients who received second-line chemotherapy for advanced PDAC [25].

Patients with homologous recombination repair deficiency (HRD) may be another indication for FOLFOX even after failure of 5-FU/LV + nal-IRI, although one patient with a germline *BRCA* mutation did not show any response in our study. Patients with a gene mutation associated with HRD, particularly germline *BRCA* mutation, are expected to respond to platinum-containing regimens because platinum-induced double-strand breaks cannot be fixed in cancer cells with HRD, resulting in cell death [26]. *BRCA* mutation status was not examined in nine patients in this study because these patients were diagnosed with contraindications for FOLFIRINOX. The prevalence of germline *BRCA* mutation was reported to be 4–7% [27–29] and that of HRD gene mutations, such as *ATM, PALB2, CHEK2, and RAD51C,* may be higher [14, 30]. HRD gene mutation was reported to be predictive of the FOLFOX regimen [14]. Next-generation sequencing using a cancer genome panel would aid in selecting patients who may be candidates for FOLFOX in the third or later lines of advanced PDAC.

Regarding safety, anaemia was the most common adverse event; however, the baseline haemoglobin level was grades 1–2 in 24 patients (56%) and the difference in haemoglobin level between baseline and worse point (Additional fig. 1-a, b), therefore, anaemia was a manageable toxicity. Therefore, it was not an obstacle to using FOLFOX as a third- or later-line treatment for patients with PDAC. Moreover, grade 1 non-haematological adverse events might have been overlooked due to the retrospective nature of this study. In addition, short PFS may underestimate the incidence and severity of peripheral sensory neuropathy (PSN), since PSN is worsen upon L-OHP dose accumulation. Therefore, patients with grade 2 PSN should be carefully treated with a FOLFOX regimen. Incidence of chemotherapy-induced nausea and vomiting was higher during FOLFOX therapy than that experienced during 5-FU/LV + nal-IRI or FOLFIRI as second-line treatment [8, 31]. This could be caused by the disease symptom itself and poorer general condition of patients in the third- or later-line treatment than that in the second-line; however, the use of maximum anti-emetic treatment, such as aprepitant, palonosetron, and dexamethasone, must be considered when administering FOLFOX as third- or later-line treatment since the incidence of nausea and vomiting was high in our study.

Our study has some limitations. First, it was retrospective study. It is preferable to evaluate the efficacy with prespecified thresholds and expectations; however, there have been little reports on OS or PFS data by best supportive care after 5-FU/LV + nal-IRI. The results of our study will help to facilitate future clinical studies to develop third-line treatment after 5-FU/LV + nal-IRI in advanced PDAC. Second, the sample size was small, especially for the multivariable analysis. Third, we had no comparator arm to evaluate the efficacy of FOLFOX treatment. Nevertheless, to the best of our knowledge, our study included the largest cohort of patients who received FOLFOX as a treatment following 5-FU/LV + nal-IRI.

## Conclusion

FOLFOX as a third- or later-line treatment following 5-FU/LV + nal-IRI was well tolerated in patients with advanced pancreatic cancer; however, its efficacy was limited. Careful patient selection, such as selection based on the CRP level, is needed.

## Supplementary Information

**Additional file 1:** **Additional fig. 1.** (a) Haemoglobin (Hb) level at baseline and worse point during the FOLFOX treatment. White and black boxes represent Hb levels at baseline and worse point, respectively. Concomitant grades 1 and 2 anaemia at baseline was observed in 12 and 12 patients, respectively. (b) Change in Haemoglobin (Hb) level from baseline to worse point during the FOLFOX treatment. Most patients showed a decrease in Hb within 2.0 g/dL, except for three patients.

## Data Availability

The datasets used and/or analysed in this study are available from the corresponding author on reasonable request. Additional informed consent and approval by Institutional Review Board is required.

## Abbreviations

PDAC: Pancreatic ductal adenocarcinoma
FOLFIRINOX: Fluorouracil (5-FU), leucovorin (LV), irinotecan, and oxaliplatin (L-OHP), as well as gemcitabine (GEM) plus nab-paclitaxel (nab-PTX)
5-FU/LV + nal-IRI: Fluorouracil, leucovorin, and nanoliposomal-irinotecan combination therapy
FOLFOX: Oxaliplatin with 5FU/LV
OS: Overall survival
PFS: Progression-free survival
CR: Complete response
PR: Partial response
SD: Stable disease
PD: Progressive disease
CI: Confidence interval
CRP: C-reactive protein
ECOG PS: Eastern Cooperative Oncology Group performance status
CEA: Carcinoembryonic antigen
CA 19–9: Carbohydrate antigen 19–9
HRD: Homologous recombination repair deficiency
