# Supplementary figures and images for: FOLFOX regimen after failure of fluorouracil and leucovorin plus nanoliposomal-irinotecan therapy for advanced pancreatic cancer: a retrospective observational study
Source: BMC Cancer. 2023 Feb 21;23:177. doi: 10.1186/s12885-023-10654-3 (PMC9945590; doi:10.1186/s12885-023-10654-3)

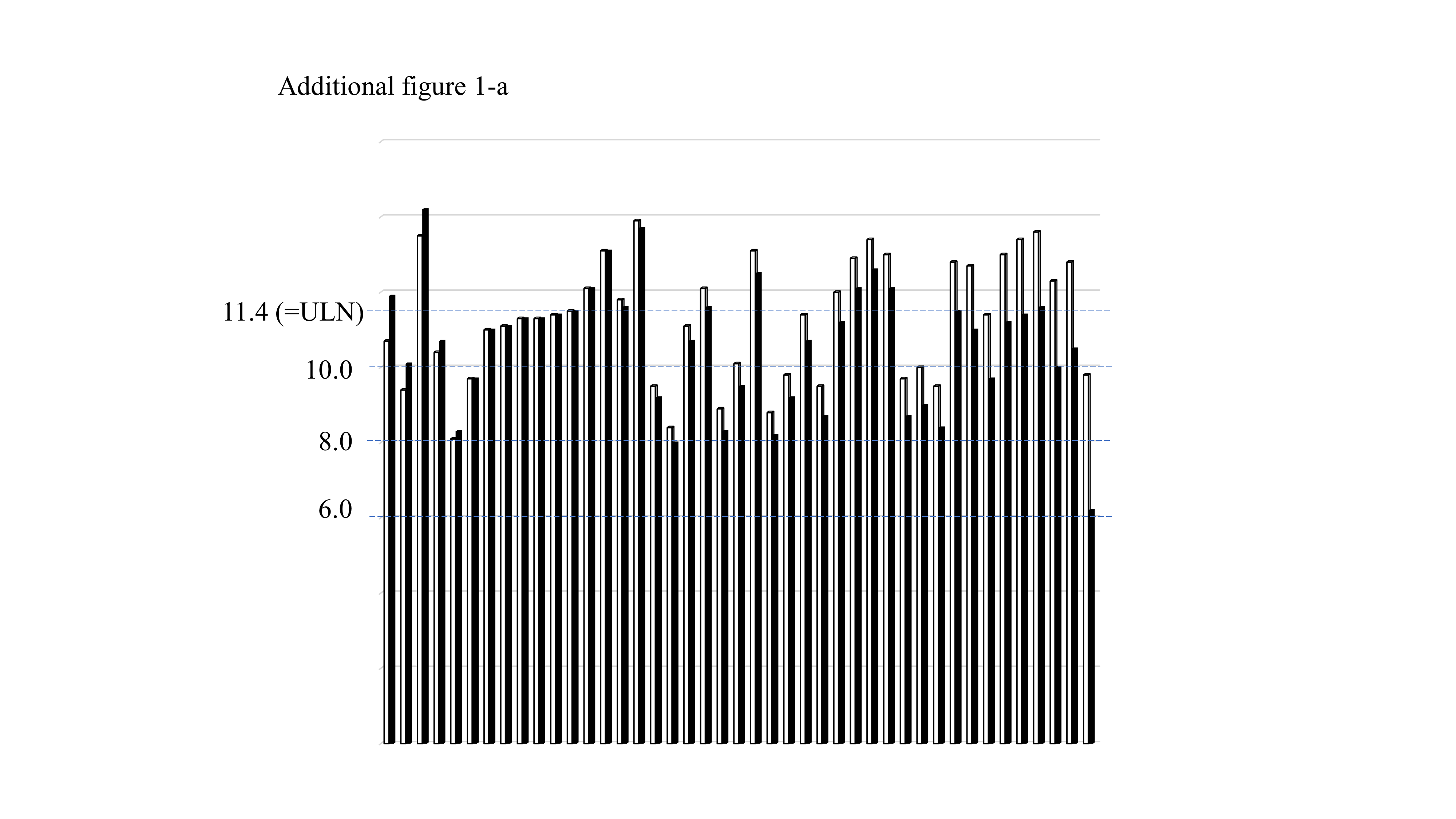

Supplement: Supplementary file 1 — Additional file 1: Additional fig. 1. (a) Haemoglobin (Hb) level at baseline and worse point during the FOLFOX treatment. White and black boxes represent Hb levels at baseline and worse point, respectively. Concomitant grades 1 and 2 anaemia at baseline was observed in 12 and 12 patients, respectively. (b) Change in Haemoglobin (Hb) level from baseline to worse point during the FOLFOX treatment. Most patients showed a decrease in Hb within 2.0 g/dL, except for three patients. [file 12885_2023_10654_MOESM1_ESM.zip › Additional figure 1-a.TIF]

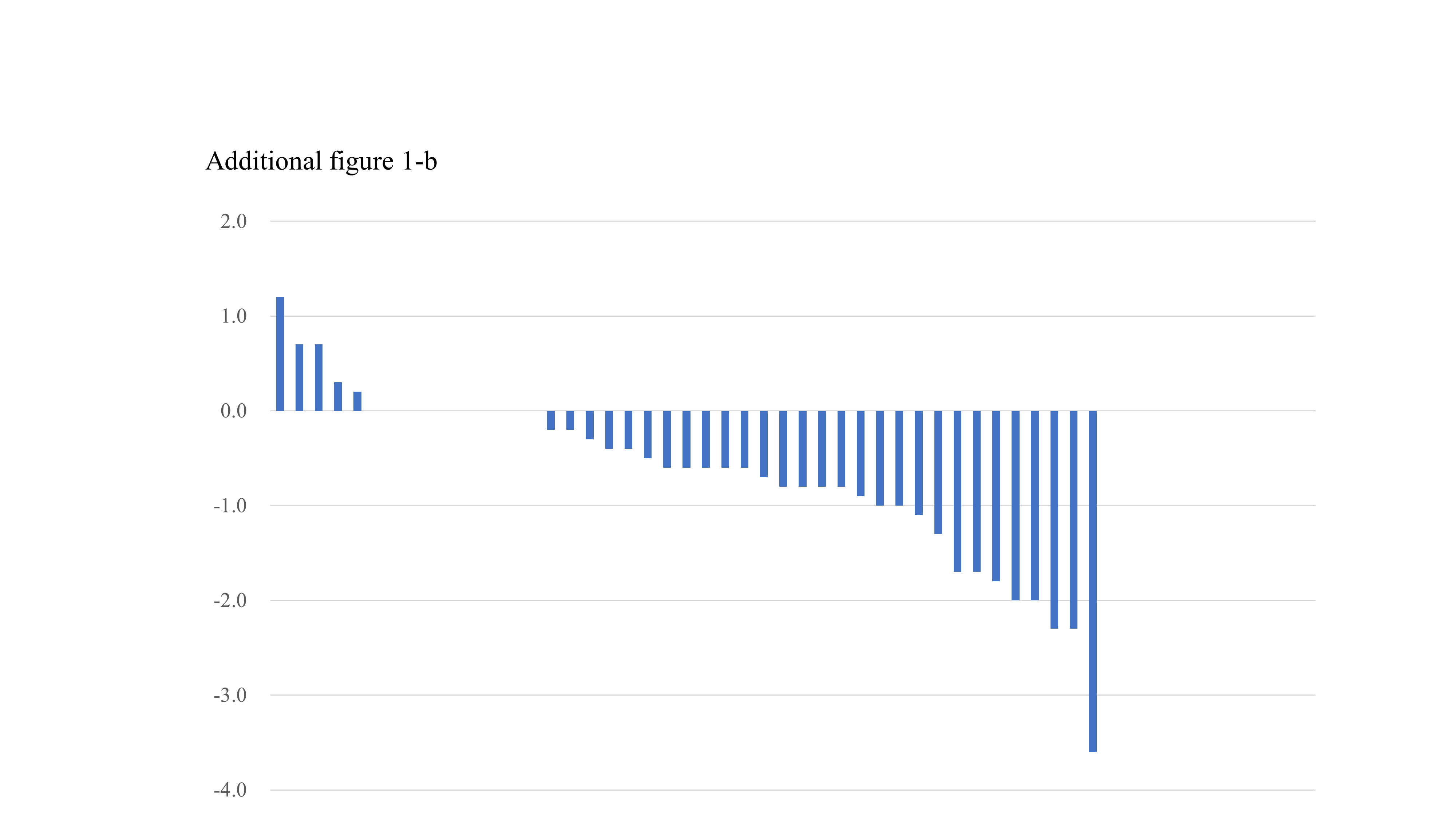

Supplement: Supplementary file 1 — Additional file 1: Additional fig. 1. (a) Haemoglobin (Hb) level at baseline and worse point during the FOLFOX treatment. White and black boxes represent Hb levels at baseline and worse point, respectively. Concomitant grades 1 and 2 anaemia at baseline was observed in 12 and 12 patients, respectively. (b) Change in Haemoglobin (Hb) level from baseline to worse point during the FOLFOX treatment. Most patients showed a decrease in Hb within 2.0 g/dL, except for three patients. [file 12885_2023_10654_MOESM1_ESM.zip › Additional figure 1-b.tif]
